# Synthesis of New Glycometronidazole Compounds With Antifungal and Antifungal Biofilm Activity

**DOI:** 10.1111/cbdd.70154

**Published:** 2025-08-11

**Authors:** Rayssa de Cassia Alves Iemini, Ana Laura Marques Trinca, Monique Dias Benedetti, Cleydson Finotti Cordeiro, Alessandro Vieira Ferreira, Amanda Latércia Tranches Dias, Ivo Santana Caldas, Jamie Anthony Hawkes, Diogo Teixeira Carvalho, Lucas Lopardi Franco

**Affiliations:** ^1^ Departamento de Alimentos e Medicamentos, Faculdade de Ciências Farmacêuticas Universidade Federal de Alfenas Alfenas Minas Gerais Brazil; ^2^ Departamento de Microbiologia e Imunologia do Instituto de Ciências Biomédicas Universidade Federal de Alfenas Alfenas Minas Gerais Brazil; ^3^ Departamento de Patologia e Parasitologia do Instituto de Ciências Biomédicas Universidade Federal de Alfenas Alfenas Minas Gerais Brazil

**Keywords:** acetylglucosamine, carbohydrates, glyco‐compounds, glycosylation, glycotriazole‐metronidazole compounds, nitroimidazole

## Abstract

Carbohydrates are well known to be one of the most abundant and structurally diverse natural organic compounds, and they are of great importance as an energy source and as structural components of cell walls in different organisms. They are involved in various biological and pathological processes, including homeostasis, cell–cell interaction, cell migration, cell development, bacterial and viral infection, inflammation, immunology, and cancer metastasis. The variety of these properties is a result of the structural diversity found in carbohydrates. The chemistry of carbohydrates involved in the diagnosis and treatment of diseases has attracted increasing attention from researchers, which is why they should be one of the main focuses in new drug discovery. This study focuses on the synthesis of new glycotriazole–metronidazole compounds as antifungal agents and antifungal biofilm agents, from the glycosylation of metronidazole with various carbohydrates (d‐glucose, d‐galactose, d‐*N*‐acetylglucosamine, and d‐lactose). Our hypothesis is that the glycosides could be taken into fungal biofilms through recognition by glycoreceptors and transporters, carrying the active residue with them. In a low‐oxygen environment, the nitro group would then undergo bioreduction leading to the formation of toxic radicals potentially resulting in the destruction or paralysis of biofilm formation—essentially functioning as a bioactive “Trojan horse.” The compounds were obtained via a click chemistry reaction using a triazole connector, and the subsequent antifungal tests showed good results for a number of compounds. In silico studies demonstrated positive data for all synthesized compounds, and, in general, they present low toxicological risks.

## Graphical abstract

Novel glyco‐triazole‐metronidazole compounds obtained via a click chemistry reaction—used as antifungal and antifungal biofilm agents. Subsequent antifungal tests showed good results for a number of compounds which are believed to essentially function as a bioactive “Trojan horse.”

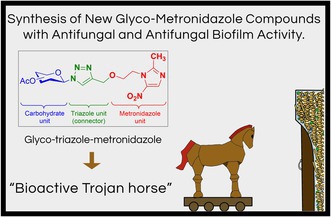

## 1 Introduction

Fungi have been a major cause of disease in humans in recent years. *Candida* species are a genus of yeasts that are responsible for the majority of human infections caused by fungal pathogens. Species of the genus *Candida* include *Candida albicans* and other emerging species such as *C. glabrata* , *C. tropicalis* , *C. parapsilosis* , *C. auris* , and *C. krusei* . Kumar et al. (2022) have also reported some rare *Candida* species such as *C. kefyr*, *C. lusitaniae* , *C. famata* , *C. guilliermondii* , *C. rugosa* , *C. nivariensis*, *C. lipolytica* , *C. bracarensis*, *C. Africana* , *C. blankie*, and *C. pulcherrima* .

*Candida albicans* colonizes soft mucous surfaces such as the oral, gastrointestinal, and genital tracts asymptomatically. However, upon disruption of the barrier integrity and/or host immune responses, the fungus can migrate through the epithelium to access deep anatomical niches and cause infections (Lopes and Lionakis 2022; Pathakumari et al. 2020; Pereira et al. 2021). The mucocutaneous surfaces mainly affected by *C. albicans* are the vaginal (vulvovaginal candidiasis), oral (oropharyngeal candidiasis), esophageal (esophageal candidiasis), and less frequently the nails (onychomycosis). Such infections are strongly related to the formation of biofilms of this species (Fritsch et al. 2022).

Biofilm formation is the preferred growth lifestyle for many microorganisms, including bacterial and fungal human pathogens. The biofilm is a strong and dynamic structure that confers a wide range of advantages to the microorganism, such as low energy and oxygen demand (dos Santos et al. 2018), with hypoxia being a typical characteristic (Hu et al. 2020).

Structurally speaking, several carbohydrates are present in the formation and maintenance of the biofilm (Bhutani et al. 2021). For example, glucose and galactose increase fungal resistance to oxidative and cationic stress, in addition to increasing their resistance to azole antifungal agents (Rodaki et al. 2009; Jamiu et al. 2021). d‐*N*‐acetylglucosamine is used for cell wall synthesis, crucial for its survival and virulence, providing protection against the external environment and structural form (dos Santos et al. 2018; Pathan and Deshpande 2022). Lactose is used by microorganisms as a carbon source which, when metabolized, provides energy and substrates necessary for biofilm growth and development (O'Neill et al. 2018; Olson et al. 2018). Furthermore, lactose can influence the expression of genes involved in cell adhesion, exopolysaccharide production, and other factors critical to biofilm formation (Awasti and Anand 2020; Carreté et al. 2018).

**FIGURE 1:**
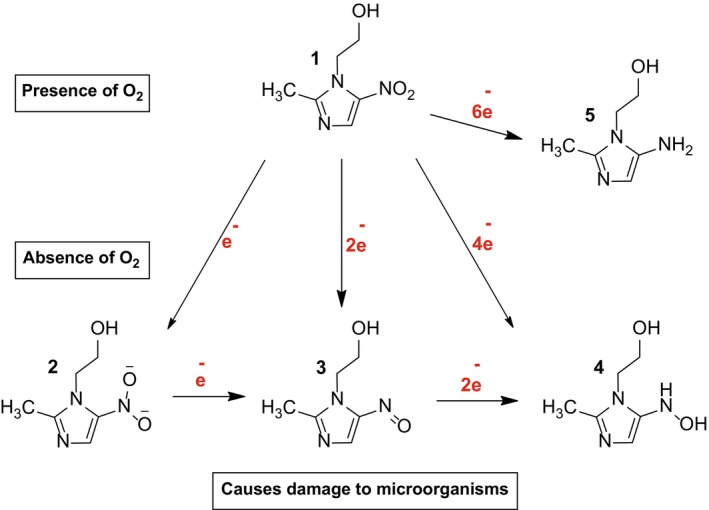
Bioreduction process of metronidazole.

Carbohydrates are one of the main focuses in the discovery of new prototypes for drug candidates, especially in the design of new glycoside‐type structural patterns containing a carbohydrate unit connected to an aglycone of therapeutic interest (Jiang et al. 2021; Magnan et al. 2021; El Malah et al. 2020). Metronidazole, which we explore in this research work, is an aglycone unit that acts as a prodrug that is activated in low‐oxygen environments by the bioreduction of the nitro group inside the target microorganism generating toxic intermediates. Some ion radicals form adducts with different molecules of the microorganism including DNA, proteins, and lipids (Miyamoto et al. 2013; Cao et al. 2017). This reduction mechanism is shown in Figure 1:

Despite this mechanism of action, metronidazole does not have relevant antifungal or antibiofilm action. Our hypothesis, raised in this work, involves the principle of using a glycometronidazole structural pattern to evaluate whether the new structural pattern will be able to increase the bioactive potential of metronidazole. This could be either by interfering in the pharmacodynamic or pharmacokinetic processes, which could lead to new glycocompounds having antifungal or antibiofilm action.

**FIGURE 2:**
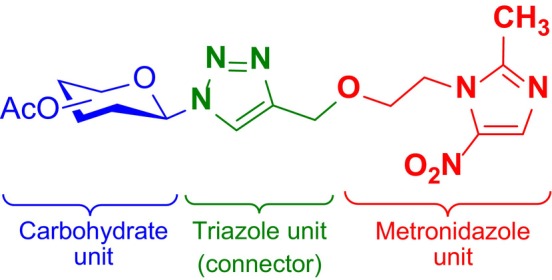
General molecular structure of the planned glycometronidazole compounds.

To explore this hypothesis, we performed nonclassical glycosylation using a “click reaction” (3 + 2) type coupling which allows beneficial synthetic access and utilizes the importance of the triazole as a pharmacophoric or auxophoric group (Kolb et al. 2001; Dixit et al. 2021; Goyard et al. 2014). This allowed the condensation of metronidazole with different carbohydrates to obtain eight new glycometronidazole compounds. The new structural pattern we obtained is recorded in Figure 2.

## 2 Methodology

Whilst there are many species of *Candida*, in this exploratory research we focused on *C. albicans* , *C. krusei* , and *C. glabrata* fungal colonies, which were cultivated and maintained in the Microbiology and Immunology Laboratory at the Universidade Federal de Alfenas, UNIFAL‐MG (Minas Gerais, Brazil). The antifungal and antifungal biofilm activity studies were also carried out in this laboratory. The cytotoxicity test was performed in the Parasitology and Pathology Laboratory at the Universidade Federal de Alfenas, UNIFAL‐MG (Minas Gerais, Brazil). All chemical reagents and solvents were purchased from Sigma Aldrich.

### 2.1 Analytical Techniques

The progress of the synthetic reactions was monitored by thin layer chromatography using silica plates with fluorescence detection (Macherey‐Nagel, DC‐Fertigfolien ALUGRAM Xtra Sil G/UV254). Purification of the synthesized compounds was performed using silica column chromatography (SCC), silica gel 60, 70‐230 mesh (Sorbiline).

### 2.2 Infrared Spectroscopy

Fourier transform infrared spectroscopy (FTIR) was performed on a Shimadzu 134 Affinity‐1 using a zinc selenide attenuated total reflectance (ATR) sampling accessory supplied by Pike Technologies (USA). Readings were taken at room temperature, 32 scans, 4.0 cm^−1^ resolution, range 4000–600 cm^−1^.

### 2.3 Nuclear Magnetic Resonance Spectroscopy

Nuclear magnetic resonance (NMR) spectroscopy was performed on a Bruker 300 spectrometer. ^1^H and COSY/HMQC experiments were recorded at 300 MHz and ^13^C experiments were recorded at 75 MHz. Tetramethylsilane (TMS) was used as an internal reference, and the samples were dissolved either in CDCl_3_ or D_2_O, depending on the compound. Chemical shifts were recorded in parts per million (ppm) based on the corresponding solvent, and abbreviations used for spin multiplicity were: s = singlet, d = doublet, t = triplet, m = multiplet.

### 2.4 Liquid Chromatography‐Mass Spectroscopy

Liquid chromatography‐mass spectrometry (LC–MS) analysis was performed on a Waters Acquity H‐Class UPLC system, comprising a quaternary solvent manager coupled to a triple quadrupole (Acquity TQD) mass spectrometer. Samples were prepared in acetonitrile, and the mobile phase was acetonitrile spiked with 0.1% formic acid. The flow rate and injection volume were 0.4 mL/min and 10.0 μL, respectively. Electrospray ionization (ESI) with positive ionization mode was considered suitable and optimized as follows: Cone voltage was 3–40 V, capillary at 3 kV and extractor at 3 V, source block temperature 120°C, desolvation line 500°C, nitrogen was used as nebulizer gas at 1000 L/h. Output signals were monitored and processed using Empower 3 software.

### 2.5 Antifungal and Antibiofilm Activity

To evaluate the effectiveness of the new compounds on biofilms of *C. albicans* , *C. krusei* , and *C. glabrata* , methodologies adapted from Ramage et al. (2001) were used. The biofilms were formed as initially described by Ramage et al. (2001) with some modifications, using RPMI cell culture medium supplemented with 2% glucose and 0.165 mol/L MOPS (3‐(*N*‐morpholino) propanesulfonic acid) as specified in the European Committee for Antimicrobial Susceptibility Testing (EUCAST) methods (Subcommittee on Antifungal Susceptibility Testing (AFST) of the ESCMID European Committee for Antimicrobial Susceptibility Testing (EUCAST) et al. 2008; Arendrup et al. 2012). The evaluation methodology adopted had been previously standardized in our research group. All substances were evaluated in serial dilution from 7500 μg/mL down to 7.3125 μg/mL.

The compounds were tested by bringing them into contact with Candida cells that had been previously adhered to the wells of the polystyrene microplate (i.e., a biofilm) and then evaluated for their ability to interfere with the metabolic activity of the biofilms. The antifungal drug fluconazole was used as a positive control, and 10 serial dilutions were tested between 2560 and 5 μg/mL at a ratio of 1:2 (v/v). The microplates were incubated at 37°C for 24 h at 75 rpm. The activity of the substances was interpreted according to their ability to reduce 50% of the metabolic activity of the biofilms formed (i.e., the IC_50_). The evaluation of the metabolic activity of the biofilm was performed through the reduction assay of the water‐soluble tetrazolium salt XTT [2,3‐bis(2‐methoxy‐4‐nitro‐5‐sulfophenyl)‐5‐((phenylamino)carbonyl)‐2H‐tetrazolium hydroxide], according to the method previously described by Ramage et al. (2001). This XTT reduction assay quantifies the capacity of the dehydrogenase enzyme, which is present in the mitochondria, to convert the XTT (yellow color) into formazan compounds (orange color). The inhibitory concentration of sessile cells was determined according to the inhibition of at least 50% of the metabolic activity of the biofilms treated with fluconazole and the evaluated substances. All assays were performed in duplicate.

### 2.6 Cytotoxicity

In order to evaluate the toxicity of the compounds and the control drugs (metronidazole and fluconazole) against Vero cells, a cytotoxicity test was performed using the protocol described by Diniz (2013) with subtle modifications. Thus, 100 μL/well of cell suspension at a concentration of 3 × 10^3^ cells/mL (in duplicate) was added to 96‐well plates, and the plates were incubated for 12 h at 37°C and 5% CO_2_. After this time, the medium was removed and replaced with 200 μL/well of a new medium containing seven different decreasing concentrations (300–4.06 μg/mL) of each of the compounds as well as of the control drugs fluconazole and metronidazole (dilution 1:2). Negative controls (DMEM medium only) and positive controls (DMEM medium and H9c2 cells) were added to each of the plates, and in order to compare the toxicity of the solvent of the samples, the DMSO control (containing dimethyl sulfoxide and medium) was used. The plates were incubated for another 48 h.

After the 48‐h incubation period, 20 μL/well of resazurin was added, and the plates were incubated again for another 12 h until the resazurin was reduced. After this period, the plates were read in a spectrophotometer at absorbances of 570 and 600 nm. From the numbers obtained, the inhibition of proliferation (%) induced by each of the compounds tested was calculated using the following equation:

$$\begin{array}{c} \%\;\text{Inhibition}\\ =100-\left[A_{570}-\left(A_{600}\times R_{0}\right)\;\text{Treated}/A_{570}-\left(A_{600}\times R_{0}\right)\;\text{Control}+\right]\times 100 \end{array}$$

where, *A*_570_ = absorbance at 570 nm; *A*_600_ = absorbance at 600 nm, and *R*_0_ = correction factor, calculated from the absorbance values of the negative control (DMEM medium without cells, with addition of resazurin) [*R*_0_ = (*A*_570_/*A*_600_) C‐]. The results of the inhibition percentage were added to the CompuSyn software to calculate the cytotoxicity data (CC_50_).

### 2.7 In Silico Study

The physicochemical, pharmacokinetic, toxicological, druglikeness, and medicinal chemistry characteristics related to the properties were determined using the OSIRIS Property Explorer program and the SwissADME Database (OSIRIS Property Explorer n.d.; Swiss Institute of Bioinformatics n.d.).

## 3 Results and Discussion

**FIGURE 3:**
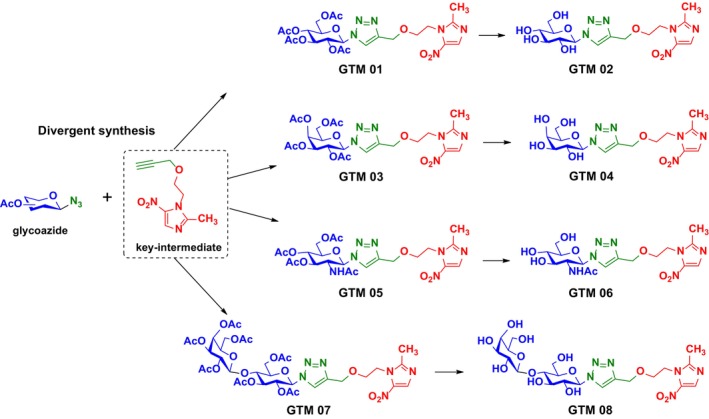
“Divergent synthesis” scheme for proposed glycosides.

**FIGURE 4:**
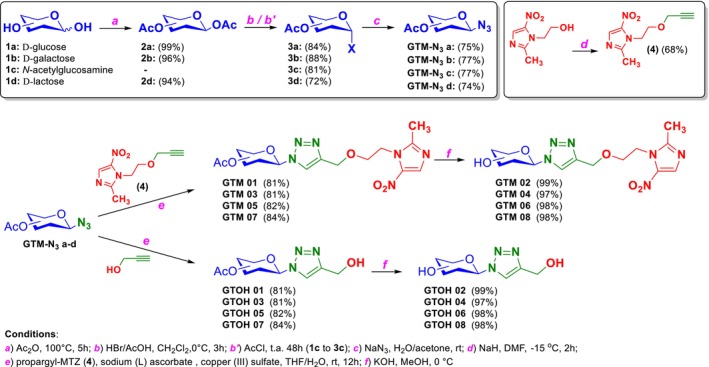
Synthesis scheme for GTM and GTOH series compounds.

The preparation of the planned glycosides involves a divergent synthesis process, by reacting a glycoazide with a key intermediate (metronidazole–propargyl), utilizing a [3 + 2] click chemistry type reaction. The general scheme is presented below:

A series of glycosides without the metronidazole unit, already reported in the literature (Baron et al. 2008; Carvalho et al. 2010; Li et al. 2011), was also planned to evaluate the importance of this metronidazole unit in the different glycotriazoles. This resulted in eight compounds of the glycotriazole–metronidazole type (referred to as the new *GTM* series) and eight of the glycotriazole type (referred to as the *GTOH* series) as shown in Figures 3 and 4.

Briefly, the carbohydrates (d‐glucose, d‐galactose, d‐*N*‐acetylglucosamine, d‐lactose) were converted into their respective peracetylated derivatives by reaction with acetic anhydride in sodium acetate and heating. They were then reacted with HBr/acetic acid to obtain the respective glycosyl halides, except for d‐*N*‐acetylglucosamine, which was directly converted into its glycosyl chloride derivative by reaction in a tube sealed with acetyl chloride. These halides were then reacted (S_N_2 mechanism) with sodium azide to obtain glycoazide intermediates. These were then either (i) coupled to the key intermediate compound (**4**) to form the **GTM** series, or (ii) coupled with propargyl alcohol to obtain the **GTOH** series. To prepare the hydroxylated derivatives, deacetylation was performed using the Zemplén Deacetylation method (Wang 2010). The general synthesis scheme is shown in Figure 4.

**FIGURE 5:**
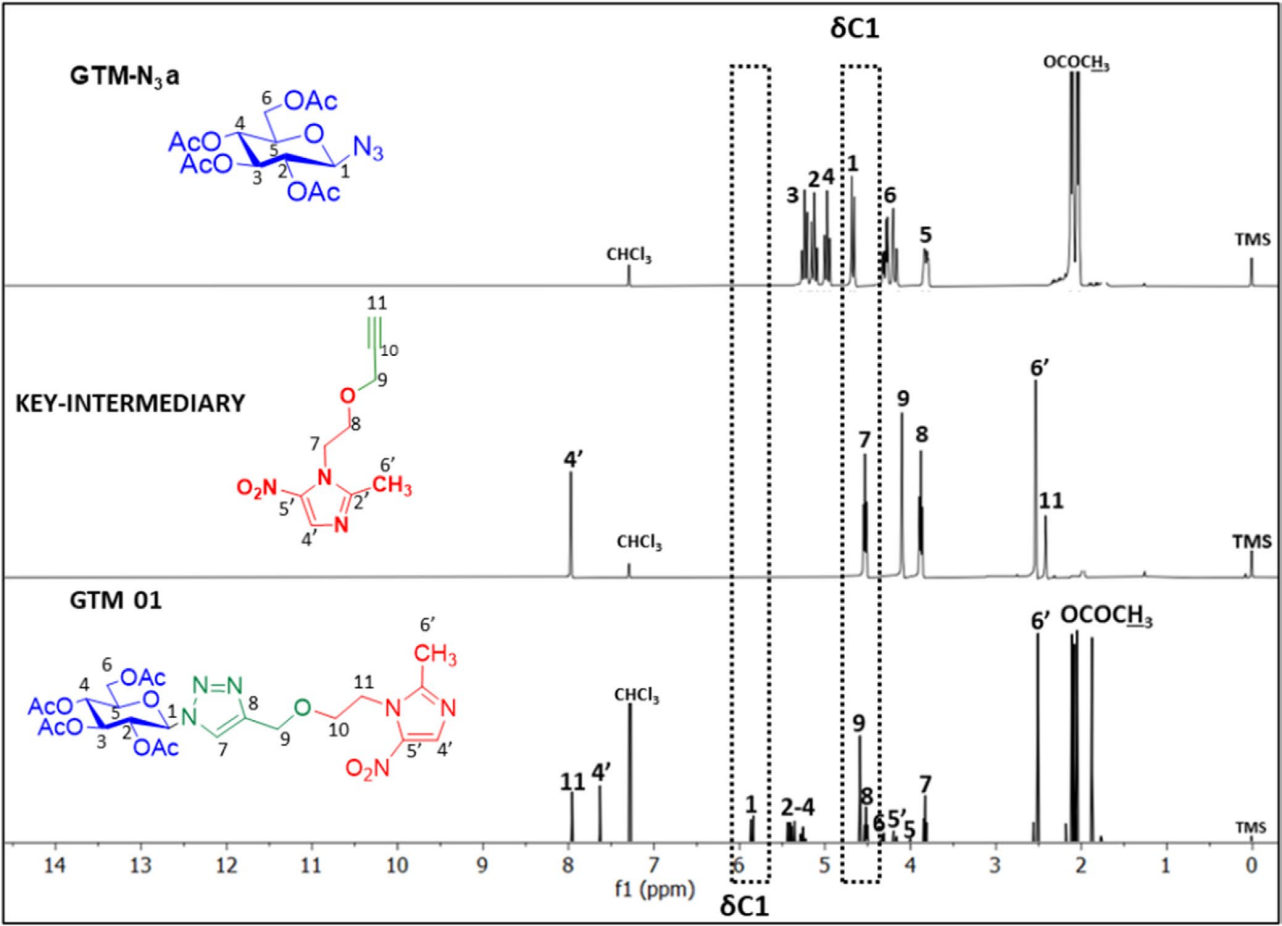
^1^H NMR spectra of compounds **GMT‐N_3_a**, **5**, and **GTM01**.

To confirm the structure of the desired glycoside compounds, FTIR (ATR), LC–MS (ESI) and NMR (^1^H and ^13^C) spectrometry were used. All spectroscopic data can be found in the Supplementary Information File (SIF), for demonstration purposes, Figure 5 shows a comparative ^1^H NMR spectrum of the glycoazide compound (**GMT‐N_3_a**), the metronidazole‐propargyl (**5**) and the resulting glycoside compound (**GTM01**). The presence of the signal at 7.45 ppm, which is characteristic of triazole hydrogen (H‐11), and the chemical shift of H‐1, which is more shielded in **GMT‐N**_**3**_ **a** (4.59 ppm) than in **GTM01** (5.85 ppm), are both consistent indications to confirm that the compound was obtained (in addition to the other characterization techniques).

### 3.1 Evaluation of Antifungal Activity of the Synthesised Glycosides

**FIGURE 6:**
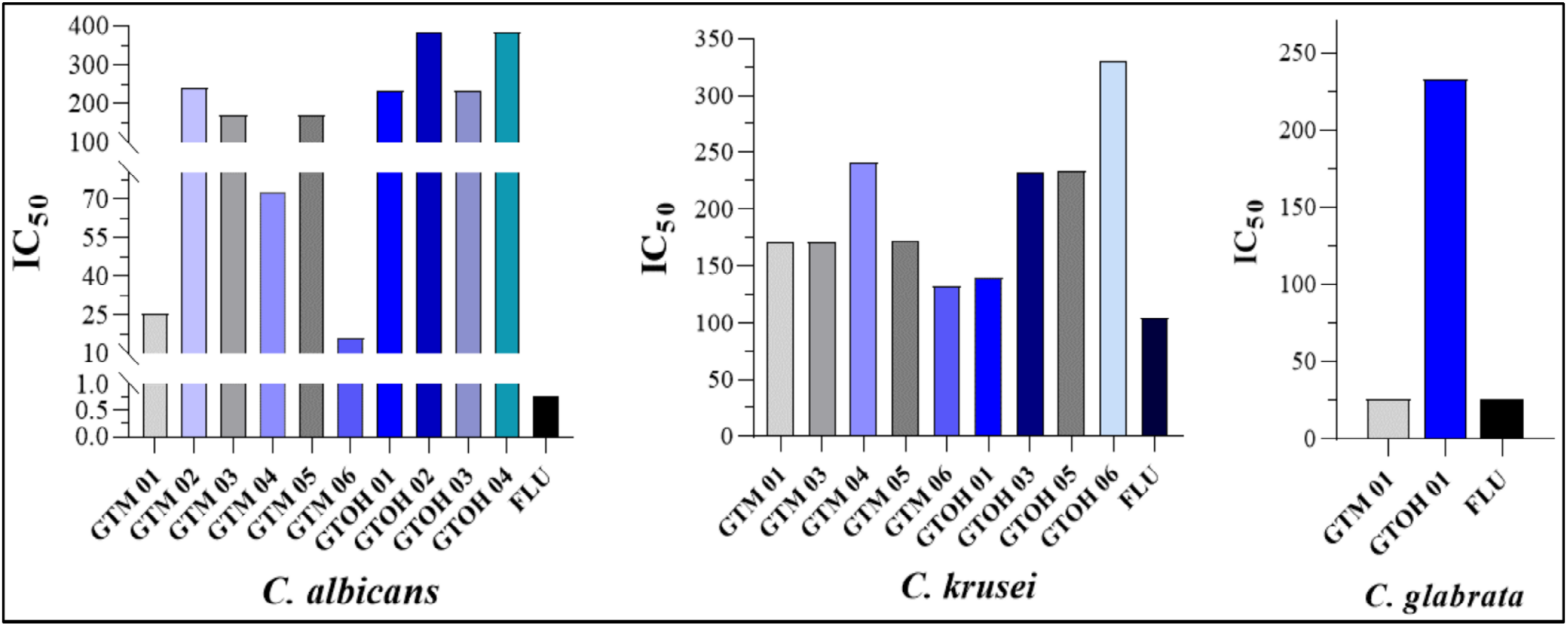
IC_50_ values of compounds against *C. albicans* , *C. krusei*, and *C. glabrata* .

The synthesized glycosides and some of their precursors were evaluated as antifungals against *C. albicans* , *C. krusei*, and *C. glabrata* as shown in Table 1 and Figure 6.

**TABLE 1:** IC_50_, CC_50_, and SI values of the compounds as antifungals (where “none” means there was no activity, and * representsthe result obtained by the lowest cytotoxic concentration tested).

Compounds	IC_50_/μM			CC_50_/μM	SI		
	*C. albicans* (S)	*C. krusei* (R)	*C. glabrata* (*S*)		*C. albicans* (S)	*C. krusei* (R)	*C. glabrata* (*S*)
**GTM01**	25.71	171.37	25.71	> 342.74*	> 13.33*	> 1.98*	> 13.33*
**GTM02**	241.44	None	None	> 482.88*	> 2.00*	None	None
**GTM03**	171.37	171.37	None	> 342.74*	> 2.00*	> 2.00*	None
**GTM04**	72.43	241.44	None	> 482.88*	> 6.67*	> 2.00*	None
**GTM05**	172.05	172.05	None	> 344.11*	> 2.00*	> 2.00*	None
**GTM06**	16.48	131.82	None	> 439.42*	> 26.66*	> 3.33*	None
**GTM07**	None	None	None	> 229.81*	None	None	None
**GTM08**	None	None	None	> 346.91*	None	None	None
**GTOH01**	233.02	139.81	233.02	> 477.17*	> 2.05*	> 3.42*	> 2.05*
**GTOH02**	383.01	None	None	> 766.02*	> 1.99*	None	None
**GTOH03**	233.02	233.02	None	> 466.04*	> 2.00*	> 2.00*	None
**GTOH04**	382.99	None	None	> 765.99*	> 2.00*	None	None
**GTOH05**	None	233.56	None	> 467.13*	None	> 2.00*	None
**GTOH06**	None	330.81	None	> 661.62*	None	> 2.00*	None
**GTOH07**	None	None	None	> 278.85*	None	None	None
**GTOH08**	None	None	None	> 407.38*	None	None	None
**GTM‐N**_**3**_ **a**	None	None	None	> 536.03*	None	None	None
**Metronidazole**	None	None	None	1110.13	None	None	None
**Fluconazole**	0.76	104.82	26.20	> 655.16*	> 862.05*	> 6.25*	> 25.01*

The results show that **GTM01** was moderately active against *C. albicans* and had antifungal action comparable to fluconazole against both *C. krusei* and *C. glabrata* . Since glucose is the preferred carbon source for *C. glabrata* , (Chew et al. 2019) we observed that only compounds containing glucose showed biological activity against it. We can also highlight that **GTM06** (derived from *N*‐acetylglucosamine) showed moderate activity against *C. albicans* and was comparable to fluconazole against *C. krusei* . We suspect that this may be related to the fact that this carbohydrate is also used for cell wall synthesis, which is crucial for the survival and virulence of the fungus and provides protection against the external environment (Pathan and Deshpande 2022). Finally, we highlight the surprising result of the glycotriazole derivative **GTOH01**, which has moderate antifungal action against *C. krusei* and *C. glabrata* .

These results show that the synthesized compounds have lower antifungal activity than expected, when compared to fluconazole, however our hypothesis was that they could be more effective in disrupting biofilms, which were then tested.

### 3.2 Evaluation of Antifungal Biofilm Activity of the Synthesized Glycosides

**FIGURE 7:**
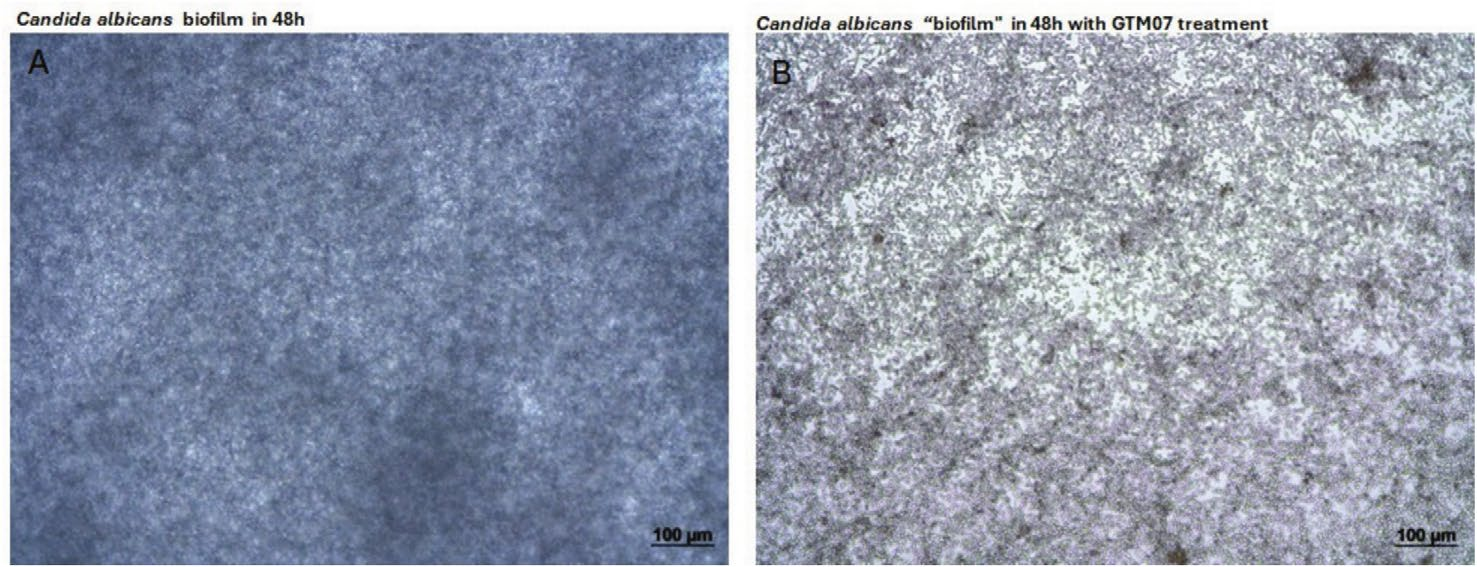
AxioCam ICc3, Zeiss optical microscopy images showing the presence and absence of extracellular matrix in *Candida albicans* biofilms: (A) control biofilm sample presenting exopolysaccharide matrix, (B) biofilm treated with **GTM07**, and not presenting an exopolysaccharide matrix.

The biofilms were formed as previously described and the synthesized compounds were tested by bringing them into contact with the *Candida* cells that had been previously adhered to the wells of the polystyrene microplate (i.e., a biofilm) and then evaluated for their ability to interfere with the biofilm development related to metabolic activity of them. As the SEM images in Figure 7a shows, *Candida* biofilm was formed as a solid matrix within 48 h of incubation. However, when new biofilm was placed in the presence of the glycoside **GTM07**, the biofilm did not mature to form a consistent exopolysaccharide matrix (Figure 7b). This simple test demonstrates the promising antibiofilm development effect of the **GTM07**.

**FIGURE 8:**
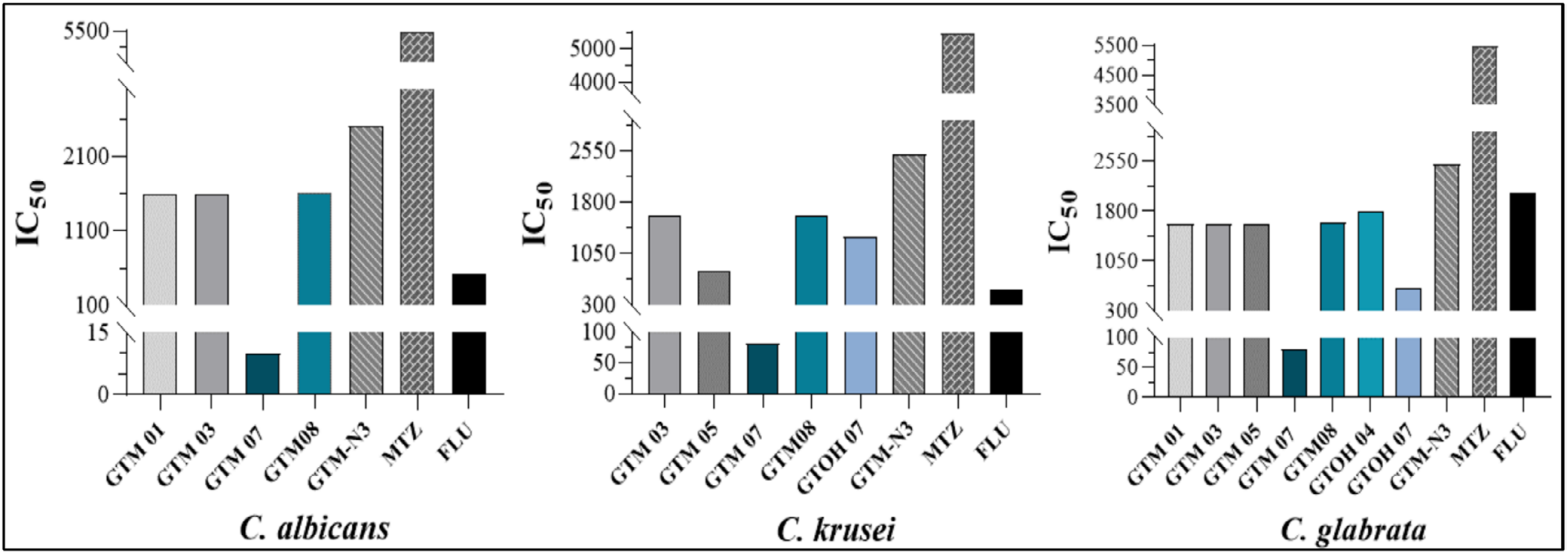
IC_50_ values demonstrating antifungal biofilm activity against *C. albicans* , *C. krusei*, and *C. glabrata*.

Figure 8 and Table 2 show the activity of the synthesized compounds against biofilms of *C. albicans* ; *C. krusei* and *C. glabrata* . Regarding the *C. albicans* biofilm; the most active compounds were **GTM01** (glycoside), **GTM03** (galactoside), **GTM07**, and **GTM08** (both lactosides). Regarding the *C. krusei* biofilm; the most active compounds were **GTM03** (galactoside), **GTM05** (*N*‐acetylglucosamide), **GTM07**, **GTM08**, and **GTOH07** (lactosides). Regarding the *C. glabrata* biofilm; a number of compounds showed activity: **GTM01** (glycoside), **GTM03** (galactoside), **GTM05** (*N*‐acetylglucosamide), **GTM07** and **GTM08** (lactosides), **GTOH04** (galactotriazole), **GTOH07** (lactotriazole).

**TABLE 2:** IC_50_ values of synthesized compounds and control drugs against *Candida* Sp. biofilms.

Compound	IC_50_/μM								
	*C. albicans* (S)	Better than control drug?		*C. krusei* (R)	Better than control drug?		*C. glabrata (S)*	Better than control drug?	
		*Mtz*	*Flu*		*Mtz*	*Flu*		*Mtz*	*Flu*
**GTM01**	1606.60	Y		3213.20	Y		1606.60	Y	Y
**GTM01**	1606.60	Y		3213.20	Y		1606.60	Y	Y
**GTM02**	18,108.07			2263.51	Y		4527.17	Y	
**GTM03**	1606.60	Y		1606.60	Y		1606.60	Y	Y
**GTM04**	3213.20	Y		3213.20	Y		3213.20	Y	
**GTM05**	6452.06			806.50	Y		1613.01	Y	Y
**GTM06**	16,478.08			4119.52	Y		8239.04		
**GTM07**	10.19	Y	Y	81.56	Y	Y	81.56	Y	Y
**GTM08**	1626.14	Y		1626.14	Y		1626.14	Y	Y
**GTOH01**	17,476.81			4369.20	Y		8738.41		
**GTOH02**	28,725.73			14,362.86			14,362.86		
**GTOH03**	8738.41			4369.20	Y		8738.41		
**GTOH04**	3590.57	Y		3590.57	Y		1795.29	Y	Y
**GTOH05**	17,517.23			17,517.23			17,517.23		
**GTOH06**	24,810.31			24,810.31			24,810.31		
**GTOH07**	2614.26	Y		1307.13	Y		653.57	Y	Y
**GTOH08**	18,410.33			4602.58	Y		9205.17		
**GTM‐N_3_**	2512.66	Y		2512.66	Y		2512.66	Y	
**Metronidazole**	5477.33			5477.33			5477.33		
**Fluconazole**	522.41			522.41			2089.65		

As the results show, a number of compounds gave better results than the metronidazole control drug against all three species of *Candida* tested: **GTM01**, **GTM03**, **GTM04**, **GTM07**, **GTM08**, **GTOH04**, and **GTOH07**. When compared to fluconazole, only **GTM07** showed better activity against all three species, however a number of compounds showed better activity than fluconazole against *C. glabrata* : **GTM01**, **GTM03**, **GTM05**, **GTM07**, **GTM08**, **GTOH04**, and **GTOH07**.

These results reinforce our hypothesis, which was that a potential increase in the antifungal action of the new entities containing a carbohydrate unit coupled to a compound with potential action in low‐oxygen concentration environments, such as metronidazole.

The main highlight in this study was the lactoside compound **GTM07**, which used the same carbohydrate that is a carbon source metabolized by these microorganisms for biofilm growth and development. In addition, the presence of lactose can influence the expression of genes involved in cell adhesion, exopolysaccharide production, and other critical factors for biofilm formation (Awasti and Anand 2020). In addition, lactose metabolism can also alter the pH of the environment, modifying the availability of other nutrients, creating conditions that can favor the formation of biofilms (Alonso et al. 2023).

Combining these factors, we are suggesting that the glycosides could be taken into the biofilm through recognition by glycoreceptors and transporters, carrying the metronidazole unit with them. In this low‐oxygen environment, the nitro group would then undergo bioreduction, leading to the formation of toxic radicals potentially resulting in the destruction or paralysis of biofilm formation—essentially functioning as a bioactive “Trojan horse.”

### 3.3 Prediction of Toxicity Risk

The toxicity of drug candidates is a limiting factor in the drug discovery process, and its determination is important in the early stages of a drug design study. In this context, computational methods that support the screening of chemical toxicity risks allow an early and rapid recognition of these properties at low costs (Kukol 2011). Using the OSIRIS Property Explorer (OSIRIS Property Explorer n.d.), the physicochemical and toxicological data of the compounds were predicted based on the chemical structure and structural fragments (Table 3). It should be noted that some of the properties calculated by the OSIRIS program do not always correspond to the actual experimental values and are used only as indicators in early development work.

**TABLE 3:** Cytotoxic and physicochemical characteristics.

Compound	Mutagenic	Tumorigenic	Irritant	Reprotoxic	CLogP	Solubility	MW	TPSA	Druglikeness	Drug score
**GTM01**	No	No	No	No	−1.54	−2.34	583.53	218.0	−3.50	0.32
**GTM02**	No	No	No	No	−3.48	−0.70	414.18	193.7	−6.78	0.43
**GTM03**	No	No	No	No	−1.54	−2.34	583.53	218.0	−3.50	0.32
**GTM04**	No	No	No	No	−3.48	−0.70	414.18	193.7	−6.78	0.43
**GTM05**	No	No	No	No	−2.02	−2.20	581.21	220.8	−4.05	0.31
**GTM06**	No	No	No	No	−3.47	−0.97	455.18	202.6	−8.18	0.40
**GTM07**	No	No	No	No	−1.57	−2.30	870.28	210.0	−3.45	0.40
**GTM08**	No	No	No	No	−3.50	−0.65	576.52	190.8	−5.98	0.48
**GTOH01**	No	No	No	No	−1.27	−2.18	429.14	165.3	−2.13	0.46
**GTOH02**	No	No	No	No	−3.21	−0.54	261.10	141.0	−5.42	0.49
**GTOH03**	No	No	No	No	−1.27	−2.18	429.14	165.3	−2.13	0.46
**GTOH04**	No	No	No	No	−3.21	−0.54	261.10	261.0	−5.42	0.49
**GTOH05**	No	No	No	No	−1.75	−2.05	428.15	168.1	−2.18	0.46
**GTOH06**	No	No	No	No	−3.20	−0.82	302.29	149.9	−6.30	0.48
**GTOH07**	No	No	No	No	−1.20	−2.01	717.22	160.3	−2.11	0.40
**GTOH08**	No	No	No	No	−3.15	−0.40	423.15	150.1	−5.50	0.50
**Metronidazole**	No	No	No	Yes	−1.05	−0.20	171.06	83.87	0.97	0.51

The predicted parameters for the control drugs are generally in line with the published experimental values, which gives confidence in the predicted values for the synthesized hybrid molecules—although complete testing is obviously required as part of the drug approval process (Kukol 2011). In the studies on the risk of toxicity, all of the compounds were shown to be safe, the C_log_P values were negative which may indicate that the compounds have greater hydrophilicity in relation to lipophilicity (i.e., they have better interaction and affinity with water) and consequently better absorption in body fluids. According to literature data, molecules with a topological polar surface area (TPSA) less than or equal to 140 Å have better oral bioavailability (Vázquez‐Rodriguez et al. 2013). The TPSA values of the compounds presented were all greater than 140 Å, indicating a possible low permeability to biological membranes and therefore low oral bioavailability. The druglikeness and drugscore parameters allow us to assess the possibility of a molecule becoming a drug (de Medeiros et al. 2020). The values in Table 1 show that all of the druglikeness values were very negative, indicating that the compounds have almost no relation to current drugs (i.e., they are new). The drugscore data with figures all below 0.50 (Silva et al. 2015).

The pharmacokinetic data of the compounds are shown in Table 4. As the data shows, the gastrointestinal absorption was low for all compounds studied, along with negative values for passage through the blood–brain barrier. *P*‐glycoprotein (P‐go_s) is characterized as an important efflux protein for drug resistance mechanisms. The compounds **GTM01**, **GTM03**, and **GTM05** act as substrates for this protein, and the efflux process (pumping the drug out of the fungal cell) causes a decrease in its concentration and subsequent effectiveness, leading to a form of resistance to these substances in vivo. Table 4 also shows the potential of the compounds for inhibiting enzymes of the cytochrome P450 superfamily. Thus, regarding the enzymes analyzed, only the inhibition of CYP2D6_i by compounds **GTOH01** and **GTOH03**, and the inhibition of CYP3A4_i by compounds **GTM01**, **GTM03**, and **GTM05** can be observed. In view of this, the inhibition of these isoforms can increase the plasma concentration of another coadministered drug that is metabolized by any of these enzymes, which can lead to this drug having an increase in its half‐life and toxicity (Matos et al. 2022).

**TABLE 4:** Pharmacokinetic data of the compounds.

Compound	Gastrointestinal absorption	BBB	P‐go_s	CYP1A2	CYP2C19	CYP2C9	CYP2D6	CYP3A4	Log Kp (cm/s)
**GTM01**	Low	No	Yes	No	No	No	Yes	Yes	−10.44
**GTM02**	Low	No	No	No	No	No	No	No	−10.65
**GTM03**	Low	No	Yes	No	No	No	Yes	Yes	−10.44
**GTM04**	Low	No	No	No	No	No	No	No	−10.65
**GTM05**	Low	No	Yes	No	No	No	No	Yes	−10.84
**GTM06**	Low	No	No	No	No	No	No	No	−10.90
**GTM07**	Low	No	No	No	No	No	No	No	−10.30
**GTM08**	Low	No	No	No	No	No	No	No	−10.59
**GTOH01**	Low	No	No	No	No	No	No	No	−10.08
**GTOH02**	Low	No	No	No	No	No	No	No	−10.30
**GTOH03**	Low	No	No	No	No	No	No	No	−10.08
**GTOH04**	Low	No	No	No	No	No	No	No	−10.30
**GTOH05**	Low	No	No	No	No	No	No	No	−10.49
**GTOH06**	Low	No	No	No	No	No	No	No	−10.55
**GTOH07**	Low	No	No	No	No	No	No	No	−10.01
**GTOH08**	Low	No	No	No	No	No	No	No	−10.20
**Metronidazole**	High	No	No	No	No	No	No	No	−7.36

### 3.4 Broader Impact and Clinical Relevance

Biofilms have been reported to be responsible for up to 80% of microbial infections in the United States of America (Nobile and Johnson 2015). They are usually attached to solid surfaces, but can also be found in other environments such as liquid–air interfaces, meaning that they can thrive in many locations, for example, mucosal surfaces and implanted medical devices. The extracellular matrix formed in a biofilm provides a protective barrier against both the host's immune system and antifungal agents (Borghi et al. 2016), which leads to recurrent and chronic infections (de Barros et al. 2020).

*Candida albicans* is known to be a fungal species that can cause infections within the human body, which are especially serious in immunocompromised patients or those with internal medical devices. *Candida* species are well known to form highly drug‐resistant biofilms in the human host, which can be very difficult to treat. *C. auris* (Satoh et al. 2009) has been proven to be highly resistant to the antifungal drugs caspofungin and anidulafungin (Dudiuk et al. 2019) and has been responsible for several outbreaks within hospitals (Ben‐Ami et al. 2017; Lockhart et al. 2017; Morales‐López et al. 2017; Nett 2019; de Jong and Hagen 2019).

It is obvious that biofilm‐based fungal infections, especially those that are presenting drug resistance, present a major clinical threat in the coming years and require significant attention. One potential pathway forward would be to use a multipronged attack, whereby current antifungal drugs are used in combination with new or emerging compounds to offer a synergistic effect.

From this exploratory study, we have found that **GTOH07** shows activity compatible with fluconazole against *C. krusei* and *C. glabrata* biofilms, which is encouraging; however, further work will include combining such compounds with current drugs to study synergistic effects to boost clinical efficiency.

## 4 Experimental

### 4.1 Synthesis of the Peracetylated Glycoside Compounds **2a**, **2b**, and **2d**

In a mortar, 27.77 mmol of the relevant carbohydrate and 48.76 mmol of sodium acetate were finely ground. The mixture was transferred to a 250 mL round‐bottom flask, and 264.47 mmol of acetic acid were added. The mixture was kept under magnetic stirring and heated to 100°C. The reaction was monitored by TLC until the starting material was consumed (~3 h). The crude product was poured into a beaker with crushed ice and kept under stirring for a further hour. The product was vacuum filtered, resulting in a yellowish paste, which was then recrystallized in absolute ethanol, resulting in white powders.

#### 4.1.1 Analysis Data

- Compound **2a**

Appearance: white powder, Yield: 99%, mp: 131.0°C–132.0°C (compared to 131°C–132°C reported by Baron et al. (2008)).

FTIR (ATR, cm^−1^): 2969 (C—H), 1738 (C=O), 1219 (ester C—O), 1034 (C—O).

- Compound **2b**

Appearance: white powder, Yield: 96%, mp: 131.0°C–132.0°C (compared to 131°C–132°C reported by Carvalho et al. (2010)).

FTIR (ATR, cm^−1^): 1736 (C=O), 1375 (aliphatic C—C), 1211 (ester C—O), 1048 (C—O).

- Compound **2d**

Appearance: white powder, Yield: 94%, mp: 176.0°C–177.2°C (compared to 176°C–177°C reported by Lis and Sharon (1993)).

FTIR (ATR, cm^−1^): 1736 (C=O), 1370 (aliphatic C—C), 1201 (ester C—O), 1032 (C—O).

### 4.2 Synthesis of the Glycosyl Halide Compounds **3a**, **3b**, and **3d**

In a 100 mL round‐bottom flask, HBr/acetic acid was prepared by the dropwise addition of 9 mL of HBr and 41 mL of acetic anhydride with an addition funnel in an ice bath with stirring. In parallel, 10.25 mmol of the peracetylated carbohydrate and 10 mL of chloroform were kept under stirring in another 100 mL flask. The HBr/acetic acid mixture was then dripped into the peracetylated carbohydrate/chloroform mixture. After the addition, the reaction was kept under stirring for 5 h in an ice bath. The reaction was monitored by tlc (hexane:ethyl acetate, 1:1). When the reaction was complete, the mixture was poured onto ice, and three extractions were performed with 20 mL of dichloromethane. The organic phase was washed with two fractions of 20 mL of saturated NaHCO_3_ solution, resulting in a viscous yellowish oil.

#### 4.2.1 Analysis Data

- Compound **3a**

Appearance: viscous yellow oil, Yield: 84%.

FTIR (ATR, cm^−1^): 1743 (C=O), 1366 (C—C), 1225 (ester C—O), 1039 (C—O), 750 (C—Br).

- Compound **3b**

Appearance: viscous yellow oil, Yield: 88%.

FTIR (ATR, cm^−1^): 1711 (C=O), 1367 (aliphatic C—C), 1223 (ester C—O), 1029 (C—O), 798 (C—Br).

- Compound **3d**

Appearance: viscous yellow oil, Yield: 72%.

FTIR (ATR, cm^−1^): 1736 (C=O), 1370 (aliphatic C—C), 1201 (ester C—O), 1032 (C—O), 885 (C—Br).

### 4.3 Synthesis of the Glucosamine Chloride Compound **3c**

To a 50 mL round‐bottom flask, 5.47 mmol of d‐*N*‐acetylglucosamine and 5 mL of acetyl chloride were added, and the mixture was kept under a sealed system (nitrogen atmosphere) under magnetic stirring at room temperature for 48 h. The system was opened and 20 mL of dichloromethane was added. Subsequently, this organic phase was washed with 20 mL of distilled water, three 15 mL aliquots of saturated NaHCO_3_ solution, and one 20 mL aliquot of saturated NaCl solution. The organic phase was dried with Na_2_SO_4_, filtered, and concentrated on a rotary evaporator. Ethyl ether was added to the resulting paste, which was filtered to obtain the pure product.

#### 4.3.1 Analysis Data

- Compound **3c**

Appearance: yellowish white solid, Yield: 81%, mp: 209.1°C–210.4°C (compared to 210°C–211°C reported by Li et al. (2011)).

FTIR (ATR, cm^−1^): 3241 (N—H), 2956 (aliphatic C—H), 1735 (C=O), 1638 (amide C=O), 1359 (aliphatic C—C), 1205 (ester C—O), 1222 (C—O), 726 (C—Cl).

### 4.4 Synthesis of Glycoazide Compounds **GTM‐N_3_a** – **GTM‐N_3_d**

To a 100 mL round‐bottom flask, 9.72 mmol of sodium azide and 5 mL of distilled water were added, and another solution containing 4.86 mmol of relevant glycosyl halide in 15 mL of acetone was added. The solution was kept under magnetic stirring at room temperature for 3 h, and the reaction was monitored by TLC (hexane:ethyl acetate, 7:3). When the starting material had been consumed, the acetone was removed under an air flow, and a white precipitate formed, which was filtered with ice‐cold water.

#### 4.4.1 Analysis Data

- Compound **GTM‐N_3_a**

Appearance: white solid, Yield: 75, mp: 128°C–129.6°C (compared to 129°C–130°C reported by Baron et al. (2008)).

FTIR (ATR, cm^−1^): 2118 (N_3_), 1743 (C=O), 1366 (aliphatic C—C), 1250 (ester C—O), 1033 (C—O).

- Compound **GTM‐N_3_a**

Appearance: white solid, Yield: 77%, mp: 129°C–129.6°C (compared to 129°C–130°C reported by Carvalho et al. (2010)).

FTIR (ATR, cm^−1^): 2120 (N_3_), 1740 (C=O), 1348 (aliphatic C—C), 1200 (ester C—O), 1032 (C—O).

- Compound **GTM‐N_3_c**

Appearance: white solid, Yield: 77%, mp: 131.5°C–132°C (compared to 130°C–132°C reported by Li et al. (2011)).

FTIR (ATR, cm^−1^): 3360 (N_3_), 2921 (aliphatic C—H), 1735 (C=O), 1661 (amide C—O), 1365 (aliphatic C—C), 1217 (ester C—O), 1034 (C—O).

- Compound **GTM‐N_3_d**

Appearance: white solid, Yield: 74%, mp: 73°C–75°C (compared to 72°C–75°C reported by Lis and Sharon (1993)).

FTIR (ATR, cm^−1^): 2110 (N_3_), 1742 (C=O), 1366 (aliphatic C—C), 1213 (ester C—O), 1036 (C—O).

### 4.5 Preparation of the Key Intermediate Compound Metronidazole‐Propargyl (**4**)

To a 25 mL round‐bottom flask, 2.92 mmol of metronidazole in 8 mL of dimethylformamide were added under magnetic stirring at 0°C. Then, 5.84 mmol of sodium hydride was added and after 5 min a solution of 2 mL of dimethylformamide containing 5.84 mmol of propargyl bromide was added. The reaction mixture was monitored by TLC until the starting material was consumed (~1 h). The resulting mixture was placed under ventilation to evaporate and remove the solvent, and the resultant crude product was purified by column chromatography (hexane 1:1 ethyl acetate).

#### 4.5.1 Analysis Data

- Compound **4**

Yield: 68%.

FTIR (ATR, cm^−1^): 3260 (C≡CH), 2134 (C≡C), 1640 (C=N or C=O), 1528 (NO_2_), 1353 N=O.

### 4.6 Synthesis of GTM and GTOH Series Compounds **1**, **3**, **5** and **7**

To a 25 mL round‐bottom flask, 0.279 mmol of the relevant glycoazide (**GTM‐N_3_a** to **GTM‐N_3_d**) was diluted in 5 mL of tetrahydrofuran, and 0.279 mmol of the key intermediate (**4**) or propargyl alcohol was added. Sodium ascorbate and copper sulfate pentahydrate were then added, and the mixture was maintained under magnetic stirring at room temperature for approximately 30 min and monitored by TLC (with ethyl acetate as solvent). The tetrahydrofuran was removed by rotary evaporator and then placed into a separating funnel. Ethyl acetate (20 mL) was then added, and liquid–liquid extraction was performed with two 20 mL portions of EDTA solution (2.5%) and one 20 mL portion of saturated NaCl solution, resulting in a white solid.

#### 4.6.1 Analysis Data

- Compound **GTM01**

IUPAC Name: (2R.3R.4S.5R.6R)‐2‐(acetoxymethyl)‐6‐(4‐((2‐(2‐methyl‐5‐nitro‐1H‐imidazol‐1‐yl)ethoxy)methyl)‐1H‐1.2.3‐triazol‐1‐yl)tetrahydro‐2H‐pyran‐3.4.5‐triyl triacetate. Appearance: brown solid, Yield: 78%, mp: 82°C–85°C.

FTIR (ATR, cm^−1^): 2922 (aliphatic C—H); 1740 (ester C=O); 1643 (C=N); 1216 (ester C—O). ^1^H NMR [300 MHz, CDCl_3_ (ppm)]: 7.94 (s, 1H, H‐7); 7.61 (s, 1H, H‐4′); 5.82 (d, 1H, *J* = 9.33 Hz, H‐1); 5.56 (m, 3H, H 2–4); 4.59 (s, 1H, H‐9); 4.53 (m, 1H, H‐10); 4.24 (m, 3H, H 5–6); 3.83 (m, 1H, H‐11); 2.501 (s, 1H, H‐12); 2.261 (m, 12H, OCOCH_3_). ^13^C NMR [75 MHz, CDCl_3_ (ppm)]: 170.68–169.07 (OCOCH_3_); 145.12 (C‐4′); 144.77 (C‐5′); 133.11 (C‐8); 86.54 (C‐1); 74.59–61.33 (C‐9, C‐6, C‐10); 46.78 (C‐11); 29.93 (C‐12); 21.49–20.23 (OCOCH_3_).

- Compound **GTM02**

IUPAC Name: (2R.3S.4S.5R.6R)‐2‐(hydroxymethyl)‐6‐(4‐((2‐(2‐methyl‐5‐nitro‐1H‐imidazol‐1‐yl)ethoxy)methyl)‐1H‐1.2.3‐triazol‐1‐yl)tetrahydro‐2H‐pyran‐3.4.5‐triol. Appearance: brown solid, Yield: 87%, mp: 280°C–281°C.

FTIR (ATR, cm^−1^): 3370 (O—H); 2922 (aliphatic C—H); 1643 (C=N); 1642 (C—O). ^1^H NMR [300 MHz, D_2_O (ppm)]: 7.93 (s, 1H, H‐7); 5.50 (d, 1H, *J* = 9.0 Hz, H‐1); 4.31 (m, 3H, H 2–4); 3.35 (s, 1H, H‐9); 4.53 (m, 4H, H‐5‐6, H‐10); 2.99 (m, 1H, H‐11); 2.73 (s, 1H, H‐12). ^13^C NMR [75 MHz, D_2_O (ppm)]: 87.36 (C‐7); 87.33 (C‐1); 78.76 (C‐2); 75.81 (C‐3); 72.21 (C‐4); 68.85 (C‐10); 62.83 (C‐9); 60.38 (C‐6); 44.63 (C‐11); 13.71 (C‐12).

- Compound **GTM03**

IUPAC Name: (2R.3S.4S.5R.6R)‐2‐(acetoxymethyl)‐6‐(4‐((2‐(2‐methyl‐5‐nitro‐1H‐imidazol‐1‐yl)ethoxy)methyl)‐1H‐1.2.3‐triazol‐1‐yl)tetrahydro‐2H‐pyran‐3.4.5‐triyl triacetate. Appearance: brown solid, Yield: 78%, mp: 82°C–85°C.

FTIR (ATR, cm^−1^): 2922 (aliphatic C—H); 1740 (ester C=O); 1643 (C=N); 1216 (ester C—O). ^1^H NMR [300 MHz, CDCl_3_ (ppm)]: 7.94 (s, 1H, H‐7); 7.61 (s, 1H, H‐4′); 5.83 (d, 1H, *J* = 9.33. H‐1); 5.56 (m, 3H, H 2–4); 4.59 (s, 1H, H‐9); 4.53 (m, 1H, H‐10); 4.24 (m, 3H, H‐1, H 5–6); 3.83 (m, 1H, H‐11); 2.51 (s, 1H, H‐12); 2.07–2.02 (m, 12H, H—OCOCH_3_). ^13^C NMR [75 MHz, CDCl_3_ (ppm)]: 170.68–169.07 (OCOCH_3_); 151.82 (C‐2′); 144.87 (C‐5′); 138.35 (C‐8); 133.06 (C‐4); 121.04 (C‐7); 86.35 (C‐1); 77.48–64.31 (C‐2‐6, C‐9‐10); 46.36 (C‐11); 20.54 (OCOCH_3_); 14.46 (C‐12).

- Compound **GTM04**

IUPAC Name: (2R.3R.4S.5R.6R)‐2‐(hydroxymethyl)‐6‐(4‐((2‐(2‐methyl‐5‐nitro‐1H‐imidazol‐1‐yl)ethoxy)methyl)‐1H‐1.2.3‐triazol‐1‐yl)tetrahydro‐2H‐pyran‐3.4.5‐triol. Appearance: brown solid, Yield: 87%, mp: 289°C–290°C.

FTIR (ATR, cm^−1^): 3343 (O—H); 1642 (C—O). ^1^H NMR [300 MHz, D_2_O (ppm)]: 7.97 (s, 1H, H‐7); 7.96 (s, 1H, H‐4′); 5.50 (d, 1H, *J* = 9.0 Hz, H‐1); 4.31 (m, 1H, H 2–4); 3.35 (s, 1H, H‐9); 4.53 (m, 4H, H‐5‐6, H‐10); 2.99 (m, 1H, H‐11); 2.73 (s, 1H, H‐12). ^13^C NMR [75 MHz, D_2_O (ppm)]: 181.50 (C‐4′); 160.62 (C‐4′) 132.79 (C‐5′); 124.06 (C‐8); 90.49 (C‐1); 87.95 (C‐2); 78.24 (C‐3); 77.17 (C‐4) 72.87 (C‐10); 70.29 (C‐9); 60.96 (C‐6); 45.88 (C‐11); 13.30 (C‐12).

- Compound **GTM05**

IUPAC Name: (2R.3S.4R.5R.6R)‐5‐acetamido‐2‐(acetoxymethyl)‐6‐(4‐((2‐(2‐methyl‐5‐nitro‐1H‐imidazol‐1‐yl)ethoxy)methyl)‐1H‐1.2.3‐triazol‐1‐yl)tetrahydro‐2H‐pyran‐3.4‐diyl diacetate. Appearance: brown solid, Yield: 87%, mp: 95°C–96°C.

FTIR (ATR, cm^−1^): 1642 (C—O); 1749 (C=O); 1230 (C—O). ^1^H NMR [300 MHz, CDCl_3_ (ppm)]: 7.98 (s, 1H, H‐7); 7.77 (s, 1H, H‐4′); 6.82 (d, 1H, *J* = 9.33. NH); 6.04 (d, 1H, *J* = 9.90 Hz, H‐1); 5.50–5.26 (m, 1H, H‐2‐4); 4.59 (s, 1H, H‐9); 4.53–4.07 (m, 4H, H‐4‐6); 3.85 (t, 1H. H‐11); 2.50 (s, 3H, NHAc); 2.12–2.06 (m, 9H, H—OCOCH_3_); 1.76 (s, 1H, H‐12). ^13^C NMR [75 MHz, CDCl_3_ (ppm)]: 170.78–169.30 (OCOCH_3_); 151.89 (C‐2′); 145.08 (C‐5′); 138.34 (C‐7); 133.35 (C‐6′); 121.91 (C‐8); 85.96 (C‐1); 75.25–72.27 (C‐9; C‐6); 70–60 (C‐2‐5); 53.52 (C‐10); 46.36 (C‐11); 29.79 (NHCOOCH_3_); 14.68 (C‐12); 21.08 (OCOCH_3_).

- Compound **GTM06**

IUPAC Name: *N*‐((2R.3R.4R.5S.6R)‐4.5‐dihydroxy‐6‐(hydroxymethyl)‐2‐(4‐((2‐(2‐methyl‐5‐nitro‐1H‐imidazol‐1‐yl)ethoxy)methyl)‐1H‐1.2.3‐triazol‐1‐yl)tetrahydro‐2H‐pyran‐3‐yl)acetamide. Appearance: brown solid, Yield: 87%, mp: 280°C–281°C.

FTIR (ATR, cm^−1^): 3335 (O—H); 1642 (amide C—O). ^1^H NMR [300 MHz, D_2_O (ppm)]: 7.98 (s, 1H, H‐7); 7.76 (s, 1H, H‐4′); 5.60 (d, 1H, *J* = 9.0 Hz, H‐1); 5.50–5.26 (m, 3H, H 2–4); 4.59 (t, 1H, *J* = 5.7 Hz, H‐9); 4.53–4.07 (m, 3H, H‐4‐6); 3.85 (t, 1H, H‐11); 2.25 (s, 1H, H‐12). ^13^C NMR [75 MHz, D_2_O (ppm)]: 173.99 (C‐7); 161.29 (C‐4′); 152.45 (C‐2′); 143.71 (C‐5′); 123.98 (C‐8); 86.32 (C‐1); 78.84 (C‐2); 73.36 (C‐3); 69.17 (C‐4); 62.76 (10); 60.39 (C‐9); 55.36 (NHCOCH_3_); 45.87 (C‐11); 13.29 (C‐12).

- Compound **GTM07**

IUPAC Name: (2R.3S.4S.5R.6S)‐2‐(acetoxymethyl)‐6‐(((2R.3R.5R.6R)‐4.5‐diacetoxy‐2‐(acetoxymethyl)‐6‐(4‐((2‐(2‐methyl‐5‐nitro‐1H‐imidazol‐1‐yl)ethoxy)methyl)‐1H‐1.2.3‐triazol‐1‐yl)tetrahydro‐2H‐pyran‐3‐yl)oxy)tetrahydro‐2H‐pyran‐3.4.5‐triyl triacetate. Appearance: brown solid, Yield: 78%, mp: 289°C–293°C.

FTIR (ATR, cm^−1^): 2926 (C—C); 1742 (ester C=O); 1266 (ester C—O); 1044 (C—O). ^1^H NMR [300 MHz, CDCl_3_ (ppm)]: 7.98 (s, 1H, H‐7); 7.55 (s, 1H, H‐4″); 5.79 (d, 1H, *J =* 9.06 Hz, H‐1′); 5.42–5.39 (m, 2H, H‐3 and H‐3′); 5.36 (m, 1H, H‐1); 5.30 (m, 1H, H‐2′); 5.26 (m, 1H, H‐2); 4.99–4.94 (m, 3H, H‐4, H‐4, H‐10); 4.55–3.60 (m, 8H, H‐4, H‐4′, H‐5, H‐5′, H‐6, H‐6, H‐9, H‐11); 2.51 (s, 1H, H‐12); 2.16–2.04 (m, 12H, H—OCOCH_3_). ^13^C NMR [75 MHz, CDCl_3_ (ppm)]: 170.40–169.11 (OCOCH_3_); 144.88 (C‐2′); 120.94 (C‐7); 101.10 (C‐1); 85.64 (C‐1′); 72.33–60.80 (C‐2‐6; C2’‐6′ e C‐9); 46.47 (C‐10); 29.69 (C‐11); 14.53 (C‐12); 20.82 (OCOCH_3_).

- Compound **GTM08**

IUPAC Name: (2S.3R.4S.5R.6R)‐2‐(((2R.3S.5R.6R)‐4.5‐dihydroxy‐2‐(hydroxymethyl)‐6‐(4‐((2‐(2‐methyl‐5‐nitro‐1H‐imidazol‐1yl)ethoxy)methyl)‐1H‐1.2.3‐triazol‐1‐yl)tetrahydro‐2H‐pyran‐3‐yl)oxy)‐6‐(hydroxymethyl)tetrahydro‐2H‐pyran‐3.4.5‐triol. Appearance: brown oil, Yield: 87%, mp: 289°C–293°C.

FTIR (ATR, cm^−1^): 3321 (OH); 2920 (C—C); 1366 (C—C); 1026 (C—O). ^1^H NMR [300 MHz, D_2_O (ppm)]: 8.18 (m, 2H, H‐4′ and H‐7); 5.61 (d, 1H. *J =* 9.0 Hz, H‐1′); 4.40–4.30 (m, 3H, H‐1, H‐9, H‐10); 3.84–3.73 (m, 5H, H‐2, H‐2′, H‐6, H‐6′, H‐11); 3.41 (s, 1H, H‐12). ^13^C NMR [75 MHz, D_2_O (ppm)]: 124.66–124.27 (C‐7, C‐8); 102.84 (C‐1); 87.13 (C‐1′); 77.65–67.38 (C‐2‐6; C‐2′‐6′); 62.83–59.74 (C‐9‐10); 47.20 (C‐11); 12.00 (C‐12).

### 4.7 Synthesis of GTM and GTOH Series Compounds **2**, **4**, **6**, and **8**

To a 25 mL round‐bottom flask, 3 mL of methanol, 0.279 mmol of the relevant peracetylated glycoside, and 1.14 mmol of KOH were added. The reaction was maintained in an ice bath with magnetic stirring for 30 min and monitored by TLC (chloroform: methanol, 1:1). At this time, Amberlite IRA 120 ion exchange resin was added and stirring was maintained for ~15 min. The resin was then filtered off, and the solvent was removed by rotary evaporator to leave a white solid.

#### 4.7.1 Analysis Data

- Compound **GTOH01**

IUPAC Name: (2R.3R.4S.5R.6R)‐2‐(acetoxymethyl)‐6‐(4‐(hydroxymethyl)‐1H‐1.2.3‐triazol‐1‐yl)tetrahydro‐2H‐pyran‐3.4.5‐triyl triacetate. Appearance: brown oil, Yield: 81%.

FTIR (ATR, cm^−1^): 3383 (OH), 3037 (aliphatic C—H), 1740 (C=O ester), 1618 (C=O), 1618 (C=C), 1496 (C—C), 1311 (ester C—O). ^1^H NMR [300 MHz, CDCl_3_ (ppm)]: 7.81 (s, 1H, H‐7); 5.90 (d, 1H, *J* = 9.0 Hz, H‐1); 5.87–5.21 (m, 3H, H 2–4); 4.77 (s, 1H, H‐9); 4.31–4.00 (m, 3H, H‐5, H‐6); 3.99 (m, 1H, H—OH); 2.06–1.85 (m, 12H, H—OCOCH_3_). ^13^C NMR [75 MHz, CDCl_3_ (ppm)]: 170.57–169.09 (C—OCOCH_3_); 148.50 (C‐8); 120.18 (C‐7); 85.68 (C‐1); 77.47 (C‐2); 77.05 (C‐3); 76.63 (C‐4); 75.03 (C‐5); 61.52 (C‐6); 56.40 (C‐9); 20.19 (C—OCOCH_3_). Which are equivalent to results reported by Baron et al. (2008).

- Compound **GTOH02**

IUPAC Name: (2R.3S.4S.5R.6R)‐2‐(hydroxymethyl)‐6‐(4‐(hydroxymethyl)‐1H‐1.2.3‐triazol‐1‐yl)tetrahydro‐2H‐pyran‐3.4.5‐triol. Appearance: brown oil, Yield: 99%.

FTIR (ATR, cm^−1^): 3369 (O—H); 3016 (C—H aliphatic); 1676 (C=C);1496 (C—C). ^1^H NMR [300 MHz, D_2_O (ppm)]: 8.03 (s, 1H, H‐7); 5.59 (d, 1H, *J* = 9.0 Hz, H‐1); 4.58 (s, 1H, OH); 4.12 (s, 4H, H‐6, H‐6′ and H‐9); 3.72–3.47 (m, 4H, H 2–5). ^13^C NMR [75 MHz, D_2_O (ppm)]: 146.99 (C‐8); 123.24 (C‐7); 87.30 (C‐1); 78.75 (C‐2); 75.77 (C‐3); 72.13 (C‐4); 68.81 (C‐5); 54.45 (C‐6); 49.76 (C‐9). Which are equivalent to results reported by Baron et al. (2008).

- Compound **GTOH03**

IUPAC Name: (2R.3S.4S.5R.6R)‐2‐(acetoxymethyl)‐6‐(4‐(hydroxymethyl)‐1H‐1.2.3‐triazol‐1‐yl)tetrahydro‐2H‐pyran‐3.4.5‐triyl triacetate. Appearance: brown oil, Yield: 81%.

FTIR (ATR, cm^−1^): 3383 (OH); 3037 (aliphatic C—H); 1740 (ester C=O); 1618 (C=C); 1496 (C—C); 1216 (ester C—O). ^1^H NMR [300 MHz, CDCl_3_ (ppm)]: 7.85 (s, 1H, H‐7); 5.86 (d, 1H, *J* = 9.0 Hz, H‐1); 5.82–5.23 (m, 3H, H 2–4); 5.22 (s, 1H, H‐9); 4.23–4.11 (m, 3H, H‐5, H‐6); 2.83 (m, 1H, OH); 2.19–1.86 (m, 12H, H—OCOCH_3_). ^13^C NMR [75 MHz CDCl_3_ (ppm)]: 170.42–169.29 (C—OCOCH_3_); 148.50 (C‐8); 120.34 (C‐7); 86.19 (C‐1); 73.96 (C‐2); 70.75 (C‐3); 67.95 (C‐4); 66.89 (C‐5); 61.23 (C‐6); 56.34 (C‐9); 20.27 (C—OCOCH_3_). Which are equivalent to results reported by Carvalho et al. (2010).

- Compound **GTOH04**

IUPAC Name: (2R.3R.4S.5R.6R)‐2‐(hydroxymethyl)‐6‐(4‐(hydroxymethyl)‐1H‐1.2.3‐triazol‐1‐yl)tetrahydro‐2H‐pyran‐3.4.5‐triol. Appearance: brown oil, Yield: 97%.

FTIR (ATR, cm^−1^): 3369 (O—H); 3016 (aliphatic C—H); 1676 (C=C); 1496 (C—C). ^1^H NMR [300 MHz, D_2_O (ppm)]: 8.07 (s, 1H, H‐7); 5.54 (d, 1H, *J* = 9.0 Hz, H‐1); 4.59 (s, 1H, OH); 4.41–4.02 (m, 4H, H‐2‐5); 3.92–3.44 (m, 4H, H‐6, H‐6′, H‐9). ^13^C NMR [75 MHz, D_2_O (ppm)]: 147.08 (C‐8); 123.03 (C‐7); 87.92 (C‐1); 78.22 (C‐2); 72.84 (C‐3); 69.61 (C‐4); 68.47 (C‐5); 60.74 (C‐6); 54.49 (C‐9). Which are equivalent to results reported by Carvalho et al. (2010).

- Compound **GTOH05**

IUPAC Name: (2R.3S.4R.5R.6R)‐5‐acetamido‐2‐(acetoxymethyl)‐6‐(4‐(hydroxymethyl)‐1H‐1.2.3‐triazol‐1‐yl)tetrahydro‐2H‐pyran‐3.4‐diyl diacetate. Appearance: brown oil, Yield: 82%.

FTIR (ATR, cm^−1^): 3401 (OH); 2925 (C—O); 2107 (C—N); 1740 (ester C=O); 1663 (amide C=O); 1377 (C=C); 1219 (C—O ester); 1055 (C—O). ^1^H NMR [300 MHz, CDCl_3_ (ppm)]: 7.81 (s, 1H, H‐7); 6.81 (d, 1H, *J* = 9.24 Hz, NHAc); 5.90 (d, 1H, *J* = 9.0 Hz, H‐1); 5.87–5.21 (m, 3H. H‐2‐4); 4.78 (s. 1H. H‐9); 4.31–3.98 (m. 3H. H‐6.5); 3.97 (s. 1H. OH); 2.05–1.86 (m. 12H. H‐ OCOCH_3_). ^13^C NMR [75 MHz, CDCl_3_ (ppm)]: 170.58–169.09 (C—OCOCH_3_); 148.50 (C‐8); 120.18 (C‐7); 85.68 (C‐1); 77.48–56.40 (C‐2‐5;6 and 9); 29.80 (NHCOOCH_3_); 20.53–20.19 (C—OCOCH_3_). Which are equivalent to results reported by Li et al. (2011).

- Compound **GTOH06**

IUPAC Name: *N*‐((2R.3R.4R.5S.6R)‐4.5‐dihydroxy‐6‐(hydroxymethyl)‐2‐(4‐(hydroxymethyl)‐1H‐1.2.3‐triazol‐1‐yl)tetrahydro‐2H‐pyran‐3‐yl)acetamide. Appearance: brown oil, Yield: 98%.

FTIR (ATR, cm^−1^): 3357 (O—H); 2918 (aliphatic C—C); 1642 (amide C—O); 1391 (C=C); 1210 (amide C—O); 1008 (C—O). ^1^H NMR [300 MHz, D_2_O (ppm)]: 7.81 (s, 1H, H‐7); 6.81 (d, 1H, *J* = 9.24 Hz, NHAc); 5.90 (d, 1H. *J* = 9.0 Hz, H‐1); 5.87–5.21 (m, 2H, H‐2‐4); 4.78 (s, 1H, H‐9); 4.31–3.98 (m, 3H, H 5–6); 3.97 (s, 1H, OH); 2.05–1.86 (m, 12H, OCOCH_3_). ^13^C NMR [75 MHz, D_2_O (ppm)]: 146.99 (C‐8); 123.25 (C‐7); 87.30 (C‐1); 78.76–60.27 (C‐2‐5); 54.46 (C‐6); 49.77 (C‐9); 29.80 (NHCOOCH_3_). Which are equivalent to results reported by Li et al. (2011).

- Compound **GTOH07**

IUPAC Name: (2R.3S.4S.5R.6S)‐2‐(acetoxymethyl)‐6‐(((2R.3R.5R.6R)‐4.5‐diacetoxy‐2‐(acetoxymethyl)‐6‐(4‐(hydroxymethyl)‐1H‐1.2.3‐triazol‐1‐yl)tetrahydro‐2H‐pyran‐3‐yl)oxy)tetrahydro‐2H‐pyran‐3.4.5‐triyl triacetate. Appearance: brown oil, Yield: 84%.

FTIR (ATR, cm^−1^): 3380 (OH); 2912 (aliphatic C—H); 1734 (ester C=O); 1367 (C—C); 1200 (C—O); 1032 (C—O). ^1^H NMR [300 MHz, CDCl_3_ (ppm)]: 7.72 (s, 1H, H‐7); 5.87 (d, 1H. *J* = 9.0 Hz, H‐1); 5.84–5.34 (m, 4H, H‐2‐3); 5.11 (s, 1H, H‐1′); 4.98 (s, 1H, H‐9); 4.97 (s, 2H, H‐6); 4.95–4.10 (m, 6H, H 4–6); 4.08 (m, 1H, H—OH); 2.15–1.86 (m, 12H, OCOCH_3_). ^13^C NMR [75 MHz, CDCl_3_ (ppm)]: 170.44–169.13 (C—OCOCH_3_); 101.10 (C‐1); 85.50 (C‐1); 75.89–56.44 (C‐2‐6; and C‐9); 20.80 (C—OCOCH_3_). Which are equivalent to results reported by Sharma et al. (2022).

- Compound **GTOH08**

IUPAC Name: (2S.3R.4S.5R.6R)‐2‐(((2R.3S.5R.6R)‐4.5‐dihydroxy‐2‐(hydroxymethyl)‐6‐(4‐(hydroxymethyl)‐1H‐1.2.3‐triazol‐1‐yl)tetrahydro‐2H‐pyran‐3‐yl)oxy)‐6‐(hydroxymethyl)tetrahydro‐2H‐pyran‐3.4.5‐triol. Appearance: brown oil, Yield: 98%.

FTIR (ATR, cm^−1^): 3305 (OH); 1567 (C=N); 1400 (C=C). ^1^H NMR [300 MHz, D_2_O (ppm)]: 8.10 (s, 1H, H‐7); 5.59 (d, 1H, *J* = 9.0 Hz, H‐1); 4.39 (d, 1H, *J* = 9.0 Hz, H‐1′); 3.80 (s, 1H, H‐3′); 3.76 (s, 1H, OH); 4.12 (m, 6H, H‐3‐6). ^13^C NMR [75 MHz, D_2_O (ppm)]: 147.18 (C‐8); 123.34 (C‐7); 102.90 (C‐1); 87.21 (C‐1′); 77.71–68.61 (C‐2‐5); 61.08 (C‐6‐6′); 54.56 (C‐9). Which are equivalent to results reported by Sharma et al. (2022).

## 5 Final Considerations

This study expressed our hypothesis regarding the effectiveness of glycometronidazole against *Candida* sp. biofilm based upon the “mechanism of action” of metronidazole, which involves bioactivation in microaerophilic or anaerobic regions. In these conditions, low‐oxygen concentration prevents the regeneration of the nitro group, leading to what is known as the “futile cycle.” This cycle is a biological phenomenon where two opposing biochemical reactions run simultaneously, resulting in a net energy loss.

This exploratory study has allowed us to suggest that carbohydrates conjugated with metronidazole may aid in its permeation through the biofilm mass, impacting its pharmacokinetics.

We were able to test our hypothesis of exploring the synthesis of a new structural pattern of glycotriazole–metronidazole (**GTM**) compounds to evaluate the antifungal and antifungal biofilm potential against three different Candida species. Our idea that a carbohydrate unit coupled to metronidazole could transform it into an antifungal and antibiofilm agent was confirmed in this work.

The positive results obtained for the **GTOH** series were useful as it can be seen that the glycotriazole pattern has interesting activity. We can highlight **GTOH01**, a glucose derivative, with moderate activity against *C. krusei* and *C. glabrata* , in addition to **GTOH07**, a lactoside derivative with activity compatible with fluconazole against *C. krusei* and *C. glabrata* biofilms, which serves as motivation for future optimization.

The highlight from the results obtained is the lactoside **GTM07**, which fits the role of being a bioactive “Trojan horse” by inhibiting the formation of all the biofilms studied.

The authors believe that this preliminary and exploratory study serves as an excellent foundation, and we propose that the scope of this research can be expanded to further investigate the antifungal and antibiofilm properties, encompassing different pathogens and combining various compounds with current drugs to study synergistic effects.

## Conflicts of Interest

The authors declare no conflicts of interest.

## Supporting information


Data S1.

## Data Availability

The data that supports the findings of this study are available in the Supporting Information of this article.
