# Supplementary material for: Synthesis of New Glycometronidazole Compounds With Antifungal and Antifungal Biofilm Activity
Source: Chem Biol Drug Des. 2025 Aug 11;106(2):e70154. doi: 10.1111/cbdd.70154 (PMC12338862; doi:10.1111/cbdd.70154)
Supplement: Supplementary file 1 — Data S1. [file CBDD-106-e70154-s001.pdf]

Supplementary Information File

Synthesis of New Glyco-Metronidazole Compounds with Antifungal and

Rayssa de Cassia Alves Iemini[1], Ana Laura Marques Trinca[1], Monique Dias Benedetti[1], Cleydson Fonitti Cordeiro[1], Alessandro Vieira Ferreira[2], Amanda Latercia Tranches Dias[2], Ivo Santana Caldas[3], Jamie Anthony Hawkes[1], Diogo Teixeira Carvalho[1], Lucas Lopardi Franco[1]*

[1] Departamento de Alimentos e Medicamentos, Faculdade de Ciências Farmacêuticas, Universidade Federal de Alfenas, Rua Gabriel Monteiro da Silva 700, Minas Gerais, 37130-001, Brazil.

[2] Departamento de Microbiologia e Imunologia do Instituto de Ciências Biomédicas, Universidade Federal de Alfenas, Rua Gabriel Monteiro da Silva 700, Minas Gerais, 37130-001, Brazil.

[3] Departamento de Patologia e Parasitologia do Instituto de Ciências Biomédicas, Ciências Farmacêuticas, Universidade Federal de Alfenas, Rua Gabriel Monteiro da Silva 700, Minas Gerais, 37130-001, Brazil.

*Corresponding author: Lucas Lopardi Franco. Email: lucas.franco@unifal-mg.edu.br

**Synthesis Routes**	GTM series	Page 2
	GTOH series	Page 2
**Infrared Spectroscopy**	GTM series	Pages 3 -6
	GTOH series	Pages 7 - 8
**^{1}H and ^{13}C Nuclear Magnetic Resonance Spectroscopy**	GTM series	Pages 9 - 16
	GTOH series	Pages 17 – 25
**LCMS Spectroscopy**	GTM series	Pages 26 - 29
